# Rupture utérine au cours d'une mole hydatiforme

**DOI:** 10.11604/pamj.2014.18.293.5055

**Published:** 2014-08-14

**Authors:** Houssine Boufettal, Naïma Samouh

**Affiliations:** 1 Service de Gynécologie-Obstétrique « C », Centre Hospitalier Universitaire Ibn Rochd de Casablanca, Maroc

**Keywords:** Rupture, utérus, mole hydatiforme, Rupture, uterus, hydatiform mole

## Image en medicine

Une patiente est âgée de 42 ans, cinq gestes, trois pares, deux avortements spontanés, était admise au service des urgences dans un tableau d'inondation péritonéale. L'anamnèse retrouvait des douleurs pelviennes, des vomissements et une notion de retards des règles de huit semaines. L'examen à l'admission retrouvait une patiente en état de collapsus avec une pression artérielle à 60-50 mmHg, une pâleur cutanéomuqueuse et une polypnée à 30 cycles par minute. Les touchers pelviens retrouvaient une sensibilité, sans masse latéro-utérine avec saignement minime ramenant des vésicules. L’échographie abdominopelvienne montrait un épanchement de grande abondance sans masse latéro-utérine, avec utérus augmenté de taille, siégeant d'images en flocons de neige en faveur d'une môle hydatiforme. L'hémogramme retrouvait une hémoglobine à 5 grammes par décilitre. Des ß-hCG plasmatiques étaient très augmentés à 1650785 mUI/mL. Un remplissage vasculaire à l'aide du sérum salé isotonique et une oxygénothérapie nasale étaient réalisés. Une laparotomie montrait un épanchement de 1000 ml fait de sang et de vésicules qui était évacué. L'exploration de la cavité abdominopelvienne retrouvait une rupture utérine en cupule du fond utérin de cinq centimètres, avec un utérus augmenté de taille mesurant 12 centimètres de longueur et huit centimètres de largeur, siégeant en intra utérin de vésicules qui était aspirées. Une hystérectomie et un lavage de la cavité abdominopelvienne au sérum salé étaient réalisés. Les suites opératoires étaient simples. L'examen anatomopathologique objectivait une môle hydatiforme complète. Les ß-hCG plasmatiques étaient négatives après huit semaines. L’évolution était favorable, avec un recul de 24 mois.

**Figure 1:**
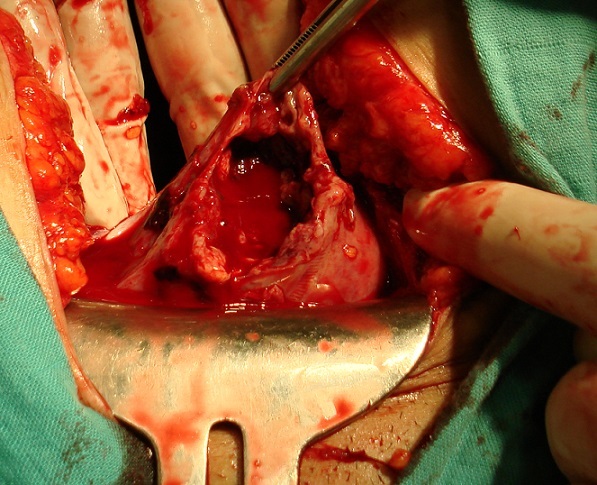
Rupture utérine en cupule du fond utérin, avec un utérus augmenté de taille siégeant en intra utérin de vésicules

